# Maternal attitudes and child-feeding practices: relationship with the BMI of Chilean children

**DOI:** 10.1186/1475-2891-8-37

**Published:** 2009-08-13

**Authors:** Christiaan Mulder, Juliana Kain, Ricardo Uauy, Jaap C Seidell

**Affiliations:** 1 Institute of Health Sciences, VU University, Amsterdam, The Netherlands; 2 Institute of Nutrition and Food Technology (INTA), University of Chile, Santiago, Chile; 3 Public Health Nutrition, London School of Hygiene and Tropical Medicine, London, UK

## Abstract

**Background:**

Chile has experienced the nutritional transition due to both social and economic progress. As a consequence, higher rates of overweight and obesity have been observed in children. In western countries, researchers have tried to determine pathways by which parents influence their children's eating behavior; up to now findings have been inconsistent. The objective of this study was to evaluate the cross-sectional and retrospective relationship between maternal attitudes and child-feeding practices and children's weight status in children who had been subject of an obesity prevention intervention for two years.

**Methods:**

In 2006, for a cross-sectional study, a random sample of 232 children (125 girls, mean age 11.91 ± 1.56 y and 107 boys mean age 11.98 ± 1.51 y) was selected from three primary schools from a small city called Casablanca. Weight and height were determined to assess their nutritional status, using body mass index (BMI) *z *scores. Child-feeding practices and attitudes were determined cross-sectionally in 2006, using the Child Feeding Questionnaire (CFQ). To analyze the relationship between trends in weight change and child-feeding practices and attitudes, BMI *z *scores of all the 232 children in 2003 were used.

**Results:**

Cross-sectionally, mothers of overweight children were significantly more concerned (P < 0.01) about their child's weight. Mothers of normal weight sons used significantly more pressure to eat (P < 0.05). Only in boys, the BMI *z *score was positively correlated with concern for child's weight (r = 0.28, P < 0.05) and negatively with pressure to eat (r = -0.21, P < 0.05). Retrospectively, the change in BMI *z *score between age 9 and 12 was positively correlated with concern for child's weight, but only in boys (r = 0.21, P < 0.05). Perceived child weight and concern for child's weight, explained 37% in boys and 45% in girls of the variance in BMI *z *score at age 12.

**Conclusion:**

Mothers of overweight children were more concerned with their children's weight; this indicated the Western negative attitude towards childhood overweight. None of the child-feeding practices were significantly correlated with a change in BMI *z *score.

## Background

The prevention and treatment of childhood overweight and obesity has become a major public health challenge. Worldwide the prevalence of overweight and obesity in children has increased rapidly during the past two decades [1,2]. Not only has the prevalence of obesity increased in developed countries, this is also the case in many countries undergoing economic transition, such as Chile [3,4]. Responsible for this trend are various changes in the social, economic and physical environment, which are elements of the nutrition transition [5,6] and are characterized by a more sedentary lifestyle, a decrease in physical activity and an increase in the consumption of foods high in fat and refined carbohydrates as well as sweetened drinks and foods that are low in fiber.

Childhood overweight is associated with elevated blood pressure, impaired glucose tolerance, insulin resistance and dyslipidaemia [7]. Moreover, long term consequences of childhood obesity are an increased risk of cardiovascular disease, type 2 diabetes and certain cancers in adulthood [8,9].

Genetic factors may play a role in the individual predisposition to obesity; however, the rapid rise in childhood overweight prevalence in genetically stable populations indicates the importance of environmental factors. Identification of modifiable environmental determinants of children's adiposity can lead to important obesity prevention practices. These determinants include parental child-feeding practices, which are thought to be significant determinants of children's weight status [10]. Research in Western countries has shown that mothers' child-feeding practices are correlated with children's food preferences [11], energy intake [10], and weight status [10]. The most studied child-feeding practices are: food restriction, pressure to eat and monitoring of food intake. These practices are considered as "controlling" child-feeding practices. Moreover, attitudes regarding the responsibility for feeding, concern for the child's weight, perceived parents weight and the perceived child's weight have been studied. However, the cross-sectional data to date has been inconsistent regarding how and if child feeding practices impact on weight. Therefore, to assign causal relationships which might explain weight change, longitudinal analyses are needed.

The objective of the present study was to evaluate maternal attitudes and child-feeding practices in relation to children's BMI, and to determine the long term effect of maternal attitudes and child-feeding practices on children's weight status, assuming that maternal attitudes and child-feeding practices persist over time [12]. The present analysis concentrated on the following two questions: do maternal attitudes and child-feeding practices differ depending on the child's weight status? Are maternal attitudes and child-feeding practices associated with child BMI z scores? We hypothesized that maternal restriction, maternal concern for child's weight and monitoring of food intake are positively correlated with children's BMI *z *scores while maternal pressure to eat is negatively correlated with these.

This research includes Chilean children who had participated in a school-based obesity prevention intervention for two years and consists of both a cross-sectional and a retrospective study. In the cross-sectional study, differences between maternal attitudes and child-feeding practices and the weight status of the children were evaluated. In the retrospective study the direction of the relationship between child-feeding practices, assessed in April 2006, and the child BMI z score, assessed in 2003 (age 9) and 2006 (age 12) was studied.

This study is unique because, to our knowledge, no other studies have examined the relationship between child-feeding practices and BMI *z *scores in Latin American children.

## Methods

### Participants

The data presented here are partly derived from the two-year school-based obesity prevention study (2003–2004) which included children of three primary schools from a small city in Chile called Casablanca [13,14]. During the intervention in 2003–2004, which is described in detail elsewhere [13], each principal informed all children, teachers and parents of the activities that were going to be carried out. Children and parents were informed that they could chose not to participate.

From the total sample size of that study at baseline in March 2003 (n = 2361, 1288 boys and 1073 girls) 981 subjects were eligible to participate in this study. Children that were included had been intervened and were in grades 1 to 5 in March 2003 (beginning of the school year). They were categorized as normal weight, overweight or obese according to the revised Centers for Disease Control Growth Charts for the United States [15] (≥ 95^th ^sex-specific percentile obese, 85^th ^≥ < 95^th ^overweight, 10^th ^≥ < 85^th ^normal weight).

From these 981 subjects, a total random sample size of 320 subjects was generated in 2006 with an equal distribution of boys (n = 160) and girls (n = 160), age groups at 2003 (young children, ages 5.75 to 8.74 y versus older children, ages 8.77 to 12.96 y) and weight status (normal weight versus overweight/obese). Selected children who had gone to other schools were replaced by randomly selected children from the 981 children of the same gender, age group and with the same weight status. Underweight children were excluded because of their small percentage. The children from 6^th ^till 8^th ^grade (mean age: 11.78 – 14.08 y) in March 2003 were excluded since they were no longer at the schools in 2006 (primary education in Chile ends in 8^th ^grade).

### Measures

Data regarding child-feeding practices were measured by self report using the Child Feeding Questionnaire (CFQ) (16). The mothers of the 320 children were requested, through a written invitation that was given to the child, to fill in the questionnaire at school. Assistance at the schools was available to help the mothers filling out the questionnaire. Data collected from relatives who filled in the questionnaires was excluded. Mothers who filled out the questionnaire received a present (1 package of pasta, 1/2 liter of salad oil and instant coffee) which was supported by a Chilean food company. Only children with a complete CFQ and subsequent anthropometric data were included in this study. Eventually, complete data was obtained from 232 children (107 boys and 125 girls); 88 children had incomplete data; 85 mothers did not fill out the questionnaire and the anthropometric data was not obtained for three children.

Permission for this study was obtained from the Educational Department of the Municipality of Casablanca and from the principals of the three primary schools.

### Measures in mothers

#### Child-feeding practices

The CFQ is a self-report measure to assess parental beliefs, attitudes and practices regarding child-feeding [16]. The questionnaire was translated into Spanish [17] and used with permission from the authors. This new version was tested for comprehension with 10 mothers from a public health center in Santiago. We assumed that these mothers were comparable to those in Casablanca, with respect to age and socioeconomic status (low).

Mother's feeding practices were measured using the 7 subscales from the CFQ. Items were scored on a 5-point Likert-type scale. Scores for all the subscale items were averaged to obtain a total score. The following subscales were used in the analysis:

##### Perceived responsibility

This measure is a 3-item subscale assessing mothers' perceptions of their responsibility for child feeding. The scale has response ratings of 1 (low feelings of responsibility) to 5 (high feelings of responsibility).

The reliability (Cronbach's α) in the present sample for this scale was 0.67.

##### Perceived parent weight

This measure is a 4-item subscale that measures parents' perceptions of their own weight status history. The scores range from 1 (markedly underweight) to 5 (markedly overweight). The reliability (Cronbach's α) in the present sample for this scale was 0.61.

##### Perceived child weight

This 6-item subscale measures parents' perceptions of their child's weight status history. The scores range from 1 (markedly underweight) to 5 (markedly overweight). The reliability (Cronbach's α) in the present sample for this scale is 0.76 and was calculated for the first 5 items. The sixth item (parents' perception of their child's weight from 6^th ^through 8^th ^grade) was applied to mothers with children from the 6^th ^trough 8^th ^grade.

##### Parents' concerns about child weight

This is a 3-item subscale that assesses parents' concerns about the child's risk of being overweight. Scores range from 1 (unconcerned) to 5 (highly concerned). The reliability (Cronbach's α) in the present sample for this scale was 0.69.

##### Restriction

This is an 8-item subscale that assesses the extent to which parents restrict their child's access to palatable foods. This measure considers restriction of both the type and amount of food. Scores range from 1 (low restriction) to 5 (high restriction). The reliability (Cronbach's α) in the present sample for this scale was 0.71.

##### Pressure to eat

This is a 4-item subscale that measures parents' tendency to pressure their children to eat by following certain behaviors such as insisting that the child finish the food on his/her plate. Scores range from 1 (low) to 5 (high) levels of pressure. The reliability (Cronbach's α) in the present sample for this scale was 0.60.

##### Monitoring

This is a 3-item subscale that assesses the extent to which parents oversee their child's consumption of sweets, snack foods and high-fat foods. Scores range from 1 (never) to 5 (always). The reliability (Cronbach's α) in the present sample for this scale was 0.75.

#### Mothers perception of own BMI and that of her child

To obtain more information regarding the weight status of the mothers, data on perceived body weight, body height and body image of the mothers was obtained. The mothers were asked to fill in their body weight and height; subsequently their own perceived BMI could be calculated. Mothers' perceived body image was obtained using the Stunkard Body Images [18]. Permission from the authors was obtained to use these figures.

In addition, mothers were asked to give their perception regarding weight and height of their children.

### Measures in Children

Children were categorized into normal and overweight/obese according to their BMI *z *score (height and weight were determined in light clothing and without shoes, measured to the nearest 0.1 cm and 0.1 kg using a Seca scale with incorporated stadiometer (Seca 720). The scale was calibrated each day. These measurements were determined by a trained public health research student and a trained health center worker from Casablanca.

### Statistical Analysis

All analyses were performed with SPSS for WINDOWS (version 10.0, SPSS Inc, Chicago). To analyze the sample for possible bias due to nonparticipation, *t *tests and Chi-square tests were used to compare children with complete data and children without complete data. Age, BMI at baseline (March 2003), BMI in 2006 and gender were compared. Previous research suggests that maternal child-feeding practices may differ between boys and girls; therefore most analyses were performed separately.

Descriptive statistics are presented as means ± SD according to weight category using the CDC reference. Overweight (p BMI 85^th ^≥ < 95^th^) and obese (p BMI ≥ 95^th^) children were combined into one group because of the small number of obese children and labeled as overweight. *T *tests for independent samples were used to detect differences in maternal feeding practices between normal weight and overweight children. Pearson correlations between child-feeding attitudes and practices, the additional perceptions and BMI z scores at age 12 (cross-sectional) and changes in BMI *z *score from age 9 to 12 were calculated.

Hierarchical, multiple-regression analysis was used to determine how much of the variance of BMI *z *scores at age 12 could be explained by maternal child-feeding attitudes and practices after adjusting for age 12 and the perceived maternal BMI.

To detect the influence of child-feeding practices on the changes of BMI *z *score between ages 9 and 12, BMI z scores were categorized into tertiles (decreased, remained unchanged or increased). Subsequently, the children were divided into two groups; increased BMI *z*, and decreased/unchanged BMI *z*. T tests for independent samples were used to detect differences between the score of the CFQ-factors in children with an increased BMI z compared with children with a decreased/stable BMI z.

Additionally, child weight status in relation with child-feeding practices was analyzed using the Mann Whitney test comparing subscales of the CFQ of children who were normal weight in 2003 and remained normal weight in 2006 with children that became overweight in 2006. This same analysis was done by comparing children who were overweight in 2003 and remained overweight in 2006 with children that became normal weight in 2006. A two-tailed α level of 0.05 was used for all statistical tests.

## Results

Student's t-tests showed no significant difference in BMI at age 9 and at age 12 between children with complete data and those with incomplete data, stratified by gender.

Girls with complete data were significantly (P < 0.05) younger than those with incomplete data (11.91 year vs. 12.54 year). A Chi-square test indicated that girls were more likely to have complete data than boys (P < 0.05). However, contingency coefficient showed that the relation between gender and having or not complete data was very weak (0.125).

Table 1 presents cross-sectional descriptive data and CFQ factors for the children, stratified by gender and weight status at age 12. Only one boy and one girl were classified as underweight and are therefore not included in table 1. In perceived responsibility for overweight boys one value was excluded for being an outlier.

**Table 1:** Descriptive statistics and CFQ-factors by gender and nutritional status at age 12

		Boys (mean ± SD)				Girls (mean ± SD)		
			
	N	Normal weight	N	Overweight	N	Normal weight	N	Overweight
Age (y)	55	11.97 ± 1.77	51	11.97 ± 1.20	68	11.84 ± 1.52	56	11.96 ± 1.62
BMI (kg/m2)	55	18.60 ± 1.73**	51	24.84 ± 2.62	68	18.84 ± 1.95**	56	24.78 ± 3.11
BMI z score	55	0.26 ± 0.52**	51	1.70 ± 0.32	68	0.24 ± 0.52**	56	1.55 ± 0.33
Perceived responsibility	55	4.44 ± 0.52	50	4.39 ± 0.56	68	4.52 ± 0.52	56	4.29 ± 0.75
Perceived parent weight category	54	3.15 ± 0.41*	50	3.31 ± 0.43	68	3.23 ± 0.46	56	3.31 ± 0.46
Perceived child weight category	54	2.96 ± 0.32**	51	3.35 ± 0.36	67	2.98 ± 0.32**	56	3.46 ± 0.39
Parents concern of child's weight	53	3.39 ± 1.15**	51	3.99 ± 0.90	67	3.47 ± 1.07**	56	4.25 ± 0.93
Restriction	55	3.22 ± 0.80	49	3.49 ± 0.71	65	3.15 ± 0.87	56	3.38 ± 0.92
Pressure to eat	55	4.16 ± 1.01*	51	3.71 ± 1.08	67	3.97 ± 1.03	55	3.90 ± 0.86
Monitoring	55	4.22 ± 1.02	51	4.15 ± 1.06	67	4.10 ± 1.09	55	4.40 ± 0.87
Perceived BMI of parents	51	27.49 ± 4.31	44	29.03 ± 4.85	53	26.51 ± 5.44	51	28.08 ± 4.59
Perceived body image	54	4.89 ± 1.62	51	5.12 ± 1.71	67	4.75 ± 1.76	55	5.13 ± 1.56
Perceived child's BMI	48	18.56 ± 3.54**	38	22.97 ± 3.20^a^	50	19.10 ± 2.74**	50	23.77 ± 4.04^a^

* Significant difference between normal weight and overweight group at P < 0.05.

** Significant difference between normal weight and overweight group at P < 0.01.

^a ^Perceived child's BMI is significantly lower than actual child's BMI (P < 0.01).

As shown in Table 1, mothers of overweight boys perceived their own weight category (P < 0.05) and that of their sons' (P < 0.01) significantly higher than mothers of normal weight boys. Mothers of overweight boys were more concerned about their sons' weight than mothers of normal weight sons (P < 0.01). Mothers of normal weight boys scored significant higher on pressure to eat (P < 0.05). Mothers of overweight girls perceived their daughters' weight significantly higher (P < 0.01) and were significantly more concerned about their daughters' weight (P < 0.01) than were mothers of normal weight girls. Overweight boys and girls were perceived as having a significantly greater BMI than were children of normal weight (P < 0.01). However, unlike mothers of children with normal weight, mothers of overweight children underestimated the BMI of their children (P < 0.01).

### Correlations between BMI *z*, CFQ-factors and additional perceptions

Table 2 presents the correlations between the CFQ-factors, perceived body image, perceived BMI of the parents, parents perceived BMI of their child, the BMI *z *score at age 12 and the change in BMI *z *score between age 9 and 12. As shown in Table 2, the BMI *z *score for boys at age 12 is significant and positively correlated with the perceived parent weight category (r = 0.27, P < 0.01), the perceived child weight category (r = 0.58, P < 0.01), weight concern (r = 0.28, P < 0.01) and the parents perceived BMI of their child (r = 0.61, P < 0.01). Pressure to eat is significant and negatively correlated with the BMI *z *score (r = -0.21, P < 0.05), whereas responsibility, restriction, monitoring, perceived body image and the perceived BMI of the parents were unrelated to BMI *z *scores at for boys at age 12.

**Table 2:** Pearson correlations between the child feeding practices and perceptions and the BMI *z *scores

	BMI *z *score at age 12		Change in BMI *z* score between age 9 to age 12	
	Boys	Girls	Boys	Girls
Perceived responsibility	-0.07	-0.22*	0.12	-0.01
Perceived parent weight category	0.27**	0.09	0.03	-0.11
Perceived child weight category	0.58**	0.64**	0.00	-0.07
Concern for child's weight	0.28**	0.38**	0.21*	0.08
Restriction	0.18	0.15	0.09	0.06
Pressure to eat	-0.21*	-0.05	0.05	0.03
Monitoring of food intake	-0.03	0.17	-0.01	0.00
Perceived body image	0.08	0.09	0.03	-0.07
Perceived BMI of parents	0.20	0.12	0.00	-0.10
Parents' perceived child's BMI	0.61**	0.74**	0.23*	0.13

* Correlation is significant at the .05 level (2-tailed).

** Correlation is significant at the .01 level (2-tailed).

The BMI *z *score for girls at age 12 is significant and negatively correlated with responsibility (r = -0.22, P < 0.05) and significant and positively with perceived child weight category (r = 0.64, P < 0.01), concern (r = 0.38, P < 0.01) and parents perceived BMI of their child (r = 0.74, P < 0.01). Perceived parent weight category, restriction, pressure to eat, monitoring, perceived body image and perceived BMI of parents were unrelated to BMI *z *scores for girls at age 12.

As presented in Table 2, the change in BMI *z *score of boys between age 9 and age 12 is significant and positively correlated with concern (r = 0.21, P < 0.05) and the perceived BMI of their child (r = 0.23, P < 0.05). This table also shows that none of the child-feeding attitudes and practices or the additional perceptions are significantly related to the change in BMI *z *score between ages 9 and 12 in girls.

### Regression models for BMI *z *scores at age 12

In Table 3 the multiple regression analysis showed that two subscales of the CFQ, perceived child weight and concern for child's weight, explained 37% of the variance in BMI *z *score of boys at age 12 and 45% of girls after adjustment for age 12 and the perceived maternal BMI. However the three controlling child-feeding practices, restriction, pressure to eat and monitoring, failed to account for any variance in BMI *z *score.

**Table 3:** Hierarchical Regression Model: Child-feeding practices and BMI *z *score at age 12

Variable entry	β	
	Final Model	R^2 ^(adjusted)
Boys		0.37
Responsibility	-0.15	
Perceived parents weight category	0.11	
Perceived child weight category	1.24**	
Concern	0.20**	
Restriction	0.06	
Pressure	-0.11	
Monitoring	-0.09	
Age 12	-0.05	
Perceived maternal BMI	0.09	
		
Girls		0.45
Responsibility	-0.06	
Perceived parents overweight	-0.04	
Perceived child overweight	1.12**	
Concern	0.18**	
Restriction	0.08	
Pressure	-0.07	
Monitoring	0.03	
Age 12	0.04	
Perceived maternal BMI	0.04	

**Significant at P < 0.01.

### Relation between CFQ subscales and changes in BMI *z *scores

As presented in Table 4, mothers of children who were overweight in 2003 and became normal weight between 2003 and 2006 were significantly less concerned about their child's weight than mothers of children who remained overweight between 2003 and 2006. Scores for the CFQ-subscales between children who were normal weight in 2003 and remained normal weight in 2006 or became overweight in 2006 did not show any significant differences. Moreover, no differences in CFQ subscale scores were found between children with an increased BMI *z *score and children with a stable/decreased BMI *z *score between ages 9 and 12 (data not shown).

**Table 4:** CFQ-scores among overweight (OW) children who remained overweight or became normal weight (NW)

		Boys & Girls together (median with 25^th ^and 75^th ^percentile)		
		
	N	OW 2003 – OW2006	N	OW2003 – NW2006
Responsibility	92	4.33 (4.00–4.92)	19	4.67 (4.00–5.00)
Perceived Parent Overweight	92	3.25 (3.00–3.50)	19	3.25 (3.00–3.50)
Perceived Child Overweight	92	3.45 (3.17–3.67)**	19	3.00 (3.00–3.20)
Concern	92	4.33 (3.67–5.00)**	19	3.67 (2.67–3.67)
Restriction	90	3.63 (3.00–4.00)	19	3.25 (2.75–3.75)
Pressure	91	4.00 (3.00–4.50)	19	4.25 (3.50–4.75)
Monitoring	92	5.00 (3.67–5.00)	19	4.67 (4.00–5.00)

**Significant at P < 0.01

## Discussion

This study describes the relationship between BMI *z *scores in a sample of Chilean schoolchildren and the parental attitudes about child-feeding and child-feeding practices. To our knowledge, this is the first study that describes this relationship in Latin-American children, although the prevalence of childhood obesity in Latin America has risen dramatically [4,19]. For example in 6 till 9 year old Brazilian children it increased three-fold (from 4.9% to 17.4%) during 1974 to 1997 [4].

The influence of ethnicity and culture on the relation between child-feeding practices and child weight status is largely unknown. Most studies on child-feeding practices and child-weight status have been done in Western countries [20-24]. Spruijt Metz et al. stated that models that describe the relation between child-feeding practices and child weight status have to be conducted separately by ethnicity [21]. Research with focus groups has shown that there are differences in cultural perceptions of child overweight and child-feeding practices [24]. However, Spruijt-Metz et al. found that ethnicity was not a significant predictor of total fat mass in white and African American children [20,21]. This finding indicates that similar mechanisms in child-feeding practices can occur across different ethnicities. Hence, we predicted a similar correlation between the BMI *z *scores and parental attitudes and child-feeding practices in Chilean children as in white and African American children.

Nearly all the Cronbach's α for the different subscales of the CFQ in the research addressed here reached the recommended value [25]. This indicates that the CFQ might be a valid tool in Latin America, but further validation studies should be conducted to confirm this.

In previous studies a positive relation has been found between BMI and restrictive practices [10,12,26] and monitoring [10]. In contrast, Brann et al. found that fathers of boys with an average BMI used more monitoring of food intake [27]. Spruijt-Metz et al. found that mother's concern for her child's weight is related to higher total fat mass in the child and that mother's pressure to eat is related to lower total fat mass [20]. Faith et al. found a similar relation between pressure to eat and lower BMI *z *scores [12]. Our results confirm these findings in the relation between concern for child's weight and BMI *z *scores and pressure to eat and BMI *z *scores. However, this cross-sectional relation between pressure to eat and BMI *z *scores was only found in boys. Another interesting difference between boys and girls in our study was that mothers of overweight girls perceived the weight of their daughters higher and were more concerned about their daughters' weight than were mothers of overweight boys, although the boys had higher BMI *z *scores. One possible interpretation is that mothers perceive overweight girls as more unhealthy and thus use more controlling child-feeding practices.

A key finding of our research, considering the correlations between the change in BMI *z *score between ages 9 and 12 and the child-feeding attitudes and practices, is that we found a positive correlation between concern on child's weight and the change in BMI *z *score in boys. However, because we only measured child-feeding practices at age 12, the direction of causality remains unclear. Probably the way in which mother and child influenced each others behavior was bidirectional. Therefore, we have to be very cautious when interpreting these findings. It appears obvious that mothers were more concerned when their children became heavier over the previous three years. This consideration appears to be even more reasonable in this specific group of mothers. Since the children had participated in an obesity prevention intervention, awareness was probably generated about the negative association between overweight and health. Hence, a higher score for concern in mothers with overweight children is expected or even might be due to the intervention. Moreover, the assumption concerning a high stability of child feeding practices, including concern, might be inappropriate considering the growing autonomy in the age-range of the children in our study. In our study, concern for child's weight was significantly correlated with the controlling child-feeding practices, restriction (r = 0.44, P < 0.01) and monitoring of food intake (r = 0.23, P < 0.05). This might have had an inverse effect which resulted in an increase in BMI *z *score between ages 9 and 12. One possible explanation for this is that the use of more controlling feeding practices, and in particular restriction, may have lead to the inability of children to self-regulate their energy intake [10,28]. However, it is probably even more plausible that a mother of an overweight 12 year old has used restriction and monitoring in response to the fact that their child has grown heavier over the previous three years. Unfortunately, because of our retrospective design, we are unable to explain the underlying mechanisms of the positive relation between restriction and weight. In a review, Faith et al. stated that parental feeding restriction, but no other feeding domain, was associated with increased child eating and weight status, though the underlying pathways remained unclear [29]. Conversely, in a recent study among infants, more use of restriction at 1 year significantly predicted lower weight at 2 years [30]. In another review it was concluded that restriction has short term and long term effects on children's intake resulting in weight gain between 5 and 11 years [31]. The inconsistency between study findings indicates that the exact causality between restrictive feeding practices and childhood overweight needs to be further investigated. The variables' covert and overt control needs to be included in these analyses. Ogden et al. stated that overt and covert control may be a useful expansion of existing ways to measure and conceptualize parental control [32]. Our findings indicate that the relationship between child's weight status and child-feeding practices and attitudes found in Chile are comparable with those found in studies conducted elsewhere.

Probably, Chile's rapid economic development has made its inhabitants follow a similar lifestyle to that found in western countries. However, the values found for the different CFQ subscales in our research are higher than those found in studies described before. A possible reason for this difference is that mothers included in this study were mostly low income women and maybe were more likely to give socially desirable answers Hence, higher scores were found on all the child-feeding subscales. Another explanation may be that the CFQ was developed to assess feeding practices in parents of preschool children. This study described child-feeding practices in adolescents. Parents of adolescents might have less influence on their children's eating habits and thus result in higher scores. Moreover, all the children in this study had a two-year intervention which may have influenced attitudes and child-feeding practices of the parents. Most probably, parents were more aware of the health complications of obesity. Unfortunately we did not determine the change in child-feeding practices and attitudes during the intervention.

Spruijt-Metz et al. found that specific child-feeding practices are equally related to total fat mass in both girls and boys [20]. However, most analyses in our study were performed separately for boys and girls. In our data we did find a difference in maternal child-feeding practices between boys and girls. Therefore, we recommend that these analyses be stratified by gender.

Participating mothers received a very basic present from a local food company. Providing these presents did not introduce selection bias since the families were not that poor and the decision to participate was probably not based on the present, but rather on personal motivation.

Our results should be considered in light of the study limitations. First, this cohort was from a small city in Chile and therefore the results cannot be extrapolated to other cities in Latin America as cultural differences might exist. Second, the present analysis did not examine measures on socioeconomic status, energy expenditure and dietary intake. Of special concern is that low income mothers seem to provide their children with a poor diet, e.g. with high amounts of high-fat products [33]. Recent published studies from Brazil and the US showed that within the context of the nutrition transition low-educated mothers face difficulties for assuring a healthy diet for their children [34,35].

However, Spruijt Metz et al. found that child-feeding practices are key behavioral variables that explained more of the variance in body fat than the intake of dietary fat [20]. Moreover, socioeconomic status failed to contribute to the regression equation for total fat mass in the same study when child-feeding practices were included [20].

## Conclusion

In children who were post-intervention, none of the maternal child-feeding practices explained a change in children's weight during a three-year follow-up period. We did find, supporting earlier results, that mothers who were more concerned about their child's weight had heavier children. Because of the retrospective design of our study, we could not determine the direction of causality between concern and the child's weight status. It is expected that mothers were more concerned when their child became heavier. Nevertheless, we must not forget that in fact it is a positive sign that heavier children's mothers were more concerned about their child's weight than mothers of normal weight children. This indicates the awareness of the negative health consequences of overweight. Further longitudinal research is needed to determine the exact direction in which mother and child influence each others behavior and what triggers their response. The results of our study showed that the Child Feeding Questionnaire could become a valid measure in other Latin American countries if studies confirm it. The purpose of further research should be to determine which child-feeding practices are modifiable and how to address these favorably. This can have important implications for obesity prevention efforts and therefore contribute to prevent the dramatic rise of childhood obesity in Latin America.

## Conflict of interest Statement

The authors declare that they have no competing interests.

## Authors' contributions

CM designed the study, collected and analysed the data and wrote the manuscript. JK assisted in the design of the study, in the data analyses and writing the manuscript. RU assisted in the data analyses and helped to draft the manuscript. JS participated in finalizing the manuscript. All authors read and approved the final manuscript.

## References

[B1] Lissau I, Overpeck MD, Ruan WJ, Due P, Holstein BE, Hediger ML (2004). Body mass index and overweight in adolescents in 13 European countries, Israel, and the United States. Arch Pediatr Adolesc Med.

[B2] Spurgeon D (2002). Childhood obesity in Canada has tripled in past 20 years. Bmj.

[B3] Kain J, Uauy R, Lera L, Taibo M, Albala C (2005). Trends in height and BMI of 6-year-old children during the nutrition transition in Chile. Obes Res.

[B4] Ebbeling CB, Pawlak DB, Ludwig DS (2002). Childhood obesity: public-health crisis, common sense cure. Lancet.

[B5] Popkin BM (2004). The nutrition transition: an overview of world patterns of change. Nutr Rev.

[B6] Popkin BM, Gordon-Larsen P (2004). The nutrition transition: worldwide obesity dynamics and their determinants. Int J Obes Relat Metab Disord.

[B7] American Diabetes Association (2000). Type 2 diabetes in children and adolescents. Pediatrics.

[B8] Must A, Strauss RS (1999). Risks and consequences of childhood and adolescent obesity. Int J Obes Relat Metab Disord.

[B9] Freedman DS, Dietz WH, Srinivasan SR, Berenson GS (1999). The relation of overweight to cardiovascular risk factors among children and adolescents: the Bogalusa Heart Study. Pediatrics.

[B10] Birch LL, Fisher JO (2000). Mothers' child-feeding practices influence daughters' eating and weight. Am J Clin Nutr.

[B11] Birch LL (1998). Psychological influences on the childhood diet. J Nutr.

[B12] Faith MS, Berkowitz RI, Stallings VA, Kerns J, Storey M, Stunkard AJ (2004). Parental feeding attitudes and styles and child body mass index: prospective analysis of a gene-environment interaction. Pediatrics.

[B13] Kain J, Uauy R, Albala, Vio F, Cerda R, Leyton B (2004). School-based obesity prevention in Chilean primary school children: methodology and evaluation of a controlled study. Int J Obes Relat Metab Disord.

[B14] Kain J, Vio F, Leyton B, Cerda R, Olivares S, Uauy R, Albala C (2005). Estrategia de Promoción de la salud en escolares de educación básica municipalizada de la comuna de Casablance, Chile [School-based health promotion intervention for primary schoolchildren from Casablanca, Chile]. Rev Chil Nutr.

[B15] Kuczmarski RJ, Ogden CL, Guo SS, Grummer-Strawn LM, Flegal KM, Mei Z, Wei R, Curtin LR, Roche AF, Johnson CL (2002). 2000 CDC Growth Charts for the United States: methods and development. Vital Health Stat 11.

[B16] Birch LL, Fisher JO, Grimm-Thomas K, Markey CN, Sawyer R, Johnson SL (2001). Confirmatory factor analysis of the Child Feeding Questionnaire: a measure of parental attitudes, beliefs and practices about child feeding and obesity proneness. Appetite.

[B17] Cull A, M S, Bjordal K (1998). EORTC Quality of life group, Translation procedure. Brussels.

[B18] Stunkard AJ, Sorensen T, Schulsinger F (1983). Use of the Danish Adoption Register for the study of obesity and thinness. Res Publ Assoc Res Nerv Ment Dis.

[B19] Martorell R, Khan LK, Hughes ML, Grummer-Strawn LM (1998). Obesity in Latin American women and children. J Nutr.

[B20] Spruijt-Metz D, Lindquist CH, Birch LL, Fisher JO, Goran MI (2002). Relation between mothers' child-feeding practices and children's adiposity. Am J Clin Nutr.

[B21] Spruijt-Metz D, Li C, Cohen E, Birch L, Goran M (2006). Longitudinal influence of mother's child-feeding practices on adiposity in children. J Pediatr.

[B22] Robinson TN, Kiernan M, Matheson DM, Haydel KF (2001). Is parental control over children's eating associated with childhood obesity? Results from a population-based sample of third graders. Obes Res.

[B23] Faith MS, Heshka S, Keller KL, Sherry B, Matz PE, Pietrobelli A, Allison DB (2003). Maternal-child feeding patterns and child body weight: findings from a population-based sample. Arch Pediatr Adolesc Med.

[B24] Sherry B, McDivitt J, Birch LL, Cook FH, Sanders S, Prish JL, Francis LA, Scanlon KS (2004). Attitudes, practices, and concerns about child feeding and child weight status among socioeconomically diverse white, Hispanic, and African-American mothers. J Am Diet Assoc.

[B25] Bland JM, Altman DG (1997). Cronbach's alpha. Bmj.

[B26] Lee Y, Mitchell DC, Smiciklas-Wright H, Birch LL (2001). Diet quality, nutrient intake, weight status, and feeding environments of girls meeting or exceeding recommendations for total dietary fat of the American Academy of Pediatrics. Pediatrics.

[B27] Brann LS, Skinner JD (2005). More controlling child-feeding practices are found among parents of boys with an average body mass index compared with parents of boys with a high body mass index. J Am Diet Assoc.

[B28] Faith MS, Kerns J (2005). Infant and child feeding practices and childhood overweight: the role of restriction. Matern Child Nutr.

[B29] Faith MS, Scanlon KS, Birch LL, Francis LA, Sherry B (2004). Parent-child feeding strategies and their relationships to child eating and weight status. Obes Res.

[B30] Farrow CV, Blissett J (2008). Controlling feeding practices: cause or consequence of early child weight?. Pediatrics.

[B31] Scaglioni S, Salvioni M, Galimberti C (2008). Influence of parental attitudes in the development of children eating behaviour. Br J Nutr.

[B32] Ogden J, Reynolds R, Smith A (2006). Expanding the concept of parental control: a role for overt and covert control in children's snacking behaviour?. Appetite.

[B33] Turrell G, Hewitt B, Patterson C, Oldenburg B, Gould T (2002). Socioeconomic differences in food purchasing behaviour and suggested implications for diet-related health promotion. J Hum Nutr Diet.

[B34] Lindsay AC, Machado MT, Sussner KM, Hardwick CK, Kerr LR, Peterson KE (2009). Brazilian mothers' beliefs, attitudes and practices related to child weight status and early feeding within the context of nutrition transition. J Biosoc Sci.

[B35] Babington L, Patel B (2008). Understanding child feeding practices of Vietnamese mothers. MCN Am J Matern Child Nurs.

